# Do all type 2 aortic dissection require emergency surgery?

**DOI:** 10.1093/jscr/rjx254

**Published:** 2018-01-23

**Authors:** Taner İyigün, Mugisha Kyaruzi, Mehmet Kaya

**Affiliations:** Istanbul Mehmet Akif Ersoy, Thoracic and Cardiovascular Surgery Training and Research Hospital, Istanbul, Turkey

## Abstract

We represent a case of asymptomatic isolated chronic ascending aortic dissection that lasted for 15 years in which a patient was only followed up with medical therapy that saved him from early surgical intervention.

## INTRODUCTION

Aortic dissection is one of the emergency and dramatic pathologies of the cardiovascular surgery. Dissection of the aorta begins with the tear of intima of the ascending or descending portion of the thoracic aorta. Moreover, the tear permits the blood to enter the aortic wall causing intramural hematoma that may progress from the side of tear to the distal part of the aorta (1, 2). Aorta dissection mostly presents many adverse events such as severe chest pain, dyspnea. In addition to these, due to high risk of mortality, surgical approach on correction of aortic dissection is usually mandatory.

Asymptomatic chronic aortic dissection may progress in a longer period and diagnosed accidentally. Diagnosing the partial aortic dissection on the ascending part of aorta will require surgical treatment even if no symptoms referring to the presence of aortic dissection. We feel that this case’s condition needs to reach a compromise on this issue because there is a lack of well-defined management that relates to the localized ascending aorta dissection, which does not extend to the distal or proximal aorta over time in many literatures.

## CASE REPORT

A 61-year-old Turkish man was referred to our center’s cardiology department due to his dyspnea and chest pain. On presentation, he was hypertensive with blood pressure of 159/85 mmHg, his heart rate was 87/min, respiratory rate was 12/min. Other systemic examinations were evaluated to be normal. The ECG records showed no changes. The transthoracic echocardiography (TTE) revealed ejection fraction of 50%, degenerative aortic and mitral valve with mild to moderate mitral insufficiency, mild tricuspid insufficiency, moderate to severe aortic insufficiency, diastolic left ventricular dysfunction grade 1, left chambers dilatation, ascending aorta and sinus valsalva dilatation of 47 mm and 46 mm, respectively.

**Figure 1:**
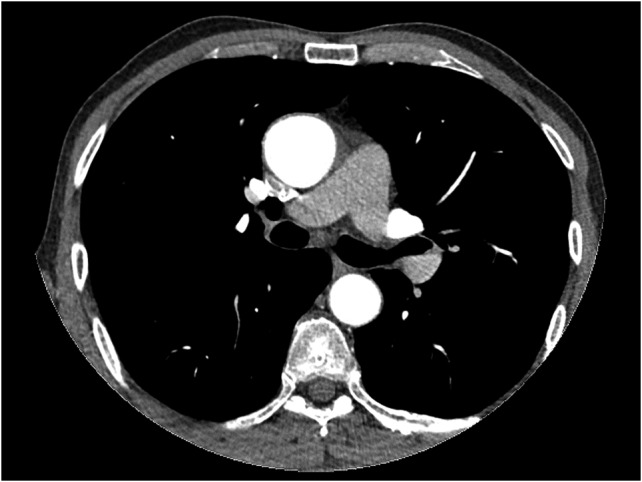
Thorax CT angiography showing the dilatation of ascending aorta of a patient before surgery on admission to our center.

Meanwhile, TEE revealed myxomatous mitral and aortic valve, severe aortic valve insufficiency, moderate to severe mitral valve insufficiency, mild tricuspid valve insufficiency, ascending aorta and sinus valsalva dilation of 46 mm. A contrast computed tomography (CT) scan was performed and revealed ascending aorta dilatation of 48.5 mm without aortic dissection and sinus valsalva of 46 mm (Fig. 1). The descending aorta appeared to be normal, measuring 26.5 mm at its widest diameter.

**Figure 2:**
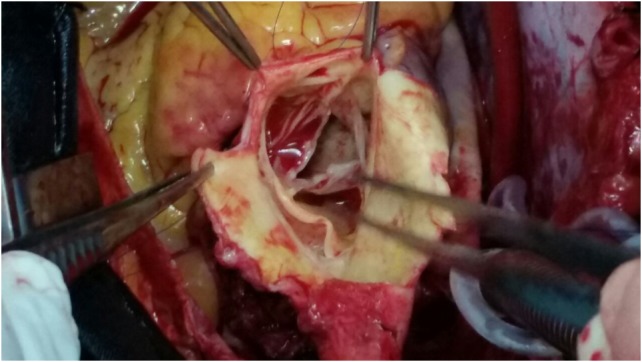
A picture that shows a chronic dissection flap of a patient during surgery.

Coronary angiography revealed a lesion on OM1 (80–90%) on its midportion where by the other coronary arteries were normal. On operation, following standard cannulation aortotomy was done where by the left main coronary artery osteal on the ascending aorta position was found to have a chronic dissection flap with dimension of 15 mm × 30 mm in size (Fig. 2). Dissection flap was resembling a mobil atheroma with free-floating tip. That the case being, modified Bentall procedure, CABG on OM1 and mitral valve replacement was perfomed.

**Figure 3:**
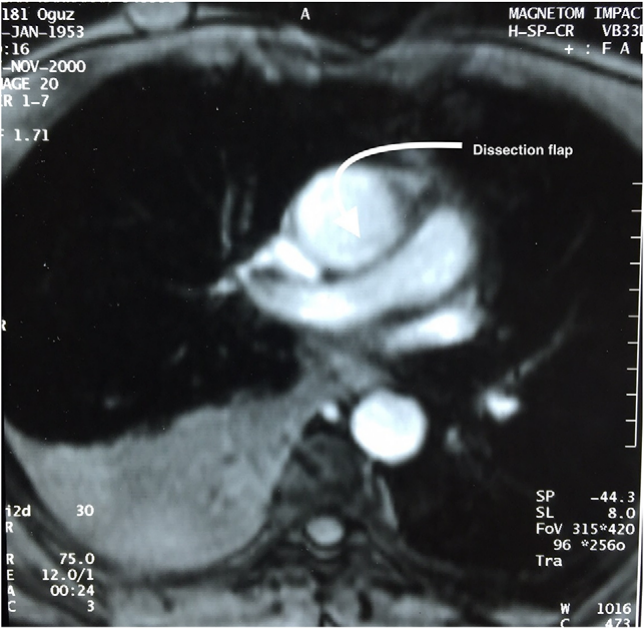
Thorax MR of a patient 15 years ago shows a dissection flap.

After observing such unexpected case (resembling type II aortic dissection) on surgery a patient was then questioned for further details on his medical history. Fifteen years ago, he was presented emergently to a cardiologist with chest pain, palpitation and sweating. On physical examination, he was hypotensive with sinus tachycardia. Both echocardiography and thorax magnetic resonance (MR) revealed pericardial effusion on posterior side of the heart (hemopericardium) with dissection flap (Fig. 3). His follow-up period was uneventful and he was then discharged from hospital with medical therapy, Echocardiography control (every 6 months) and thorax CT control (every 1 year). The last thorax CT (that we took in our clinic did not demostrate dissection flap (Fig. 1). Both imagings of Figs 1 and 3 were avaluated under the same radyologic level for accuracy.

## DISCUSSION

Aortic dissection develops when a tear in the inner wall of the aorta causes blood to flow between the layers of the wall of the aorta, causing the layers apart (1). Aortic dissection occurs more common in patients with a history of high-blood pressure that affect blood vessel wall integrity (3). Aortic dissection is a cardiovascular emergency and can quickly lead to death, even with optimal treatment, due to insufficiency blood supply to other organs and sometimes rupture of the aorta. In our case, a patient had a localized aortic dissection resembling that of chronic aortic dissection type II that lasted for 15 years without showing any symptom. Our patient had also hypertensive with pericardial and pleural effusion that may indicate a ruptured aorta. Due to high risk of rupture and being accompanied with other cardiac pathologies (mitral valve insufficiency and coronary lesion), modified Bentall procedure was planned in concordant with coronary artery bypass and mitral valve replacement.

Aortic dissection presents with severe chest pain, acute hemodynamic instability, absence or unilateral peripheral pulses, neurologic complications and aortic insufficiency (2). However, our patient was presented to our clinic with chest pain and dyspnea that may have been related to a coronary lesion and valve insufficiency. He did not have symptoms related to aortic dissection though he was under hypertensive therapy.

Transthoracic two-dimensional echocardiography (TTE) is very effective in evaluating the aortic root, but the mid and distal ascending aorta, aortic arch and descending aorta are not seen. It also provides important information on aortic valvular regurgitation, and the function and dimensions of the left ventricle (4). Our patient was diagnosed with TTE that revealed only the dilatation of aorta without dissection. For further evaluation contast thorax CT (thorax CT) was performed in our case and aortic dilatation was observed but dissection was not seen on his last thorax CT scan. This may be due to that isolated aortic dissection flap may have been endothelized within that period of fifteen years since then. However Thorax CT is the most frequent first imaging modality performed, with very high sensitivity and specificity (5). Conversly, this could have been a sporadic case that we could have reported.

Asymptomatic localized aortic dissection is a rare condition that may require early surgical intervention when diagnosed or followed up with medication depending on the underlying cause or predisposition to rupture. Our patient was diagnosed with other medical heart condition such as aortic dilatation. This may have been a condition experienced in 15 years in relation to a patient’s medical history under medical therapy.

In recent years, these type of patients with isolated asecending aortic dissection are referred either for emergency surgery or elective surgery depending on hemodynamic condition. Our preference and recommendation in these kinds of cases are to perform early surgery to be able to avoid adverse events due to aortic dissection.
